# A qualitative exploration of mental health first aid training in underserved communities facing repeated extreme weather events

**DOI:** 10.3389/fpsyt.2025.1709262

**Published:** 2026-01-12

**Authors:** Omolola E. Adepoju, Laura De la Roche, Carlos G. Fuentes, Mary Tipton, Yahaira Suchil

**Affiliations:** 1 Department of Health Systems and Population Health Sciences, University of Houston, Houston, TX, United States; 2 Humana Integrated Health Systems Sciences Institute, University of Houston, Houston, TX, United States

**Keywords:** community, disasters, environmental health, mental health, underserved and unserved populations

## Abstract

**Background:**

Successive extreme weather events, including hurricanes and floods, have been reported to negatively impact mental health. Mental Health First Aid (MHFA) training is an evidence-based approach to improve participants’ knowledge, attitudes, and confidence in recognizing, approaching, and supporting individuals experiencing mental health challenges. Investigations have supported its perceived acceptability and utility within professional and health-care populations; however, the appropriateness and applicability of this training in underserved populations in disaster-prone areas are unknown.

**Methods:**

Eligible participants self-described as residing in disaster-prone areas and participated in MHFA training between February and September, 2025 in Houston, Texas. Individual semi-structured interviews were conducted to assess perceptions of the appropriateness and utility of the MHFA training for disaster preparedness, how participants perceived mental health, and access to available resources 6-8 weeks after the MHFA training completion. Mental health support measures were used to assess the support that participants self-reported they provided to others after completing the MHFA training.

**Results:**

Most participants (63.6%) reported knowing someone who experienced a mental health problem in the past year. Supportive behaviors were common, with over 85% reporting evidence-based responses (e.g., asking about self-harm, active listening, conveying hope, and offering resources). Reflexive thematic analysis resulted in three primary themes: (1) awareness and impact of mental health struggles and the role of the community; (2) understanding the realities of mental healthcare resource access and necessary steps to increase uptake; and (3) community residents indicated the MHFA training was useful and appropriate for disaster preparedness. The findings highlight the usefulness of MHFA trainings in underserved communities that are affected by successive disasters.

**Implications:**

The results suggest that MHFA training meaningfully improved participants’ recognition of mental health challenges, confidence in offering support, and awareness of available resources, even within communities facing repeated disaster exposure. These findings also indicate that MHFA may strengthen community capacity for the early identification and response to mental health needs by equipping residents with practical skills that they actively apply in real-world situations following the training.

## Introduction

Underserved communities in disaster-prone areas are at an increased risk of experiencing mental health challenges and crises. Recognizing this, several community-based approaches for psychosocial support during emergencies and disasters have been suggested, including the Inter-Agency Standing Committee (IASC) guidelines (1) on Mental Health and Psychosocial support in Emergency settings, the Psychological First Aid model (2), and the Crisis Counseling Assistance and Training Program (CCP) (3), among others. These approaches offer a framework for providing appropriate mental health care during a crisis, with an emphasis on integrated support systems that consist of community, health services, and social services (4, 5). Recently, Mental Health First Aid (MHFA) training has gained prominence as an evidence-based approach to improve participants’ knowledge, attitudes, and confidence in recognizing, approaching, and supporting individuals experiencing mental health challenges (6).

Globally, nearly half of the world’s population is affected by mental illness, which has significant impacts on self-esteem, relationships, and daily functioning (7). In the United States, mental health conditions are a leading cause of disability, and more than 22% of adults experience a diagnosable mental illness each year, with a prevalence rate higher than that of cancer, diabetes, or heart disease (8, 9). However, access to professional care is often limited; nearly half of U.S. counties have no psychiatrist, and many communities face long wait times, high treatment costs, and fragmented insurance coverage (10). Stigma and financial hardship further discourage help-seeking (11).

Against this backdrop, MHFA provides a critical community-based solution by equipping laypeople to recognize symptoms, provide initial support, and encourage professional care for various mental health situations, including psychosis, panic attacks, suicidal thoughts, and substance use (12). This training is based on the ALGEE framework, which teaches trainees to approach potential crises with the following steps: 1. Assess for risk, 2. Listen, 3. Give reassurance, 4. Encourage appropriate professional help, 5. Encourage self-care (13). Evidence shows that MHFA training improves mental health literacy, increases confidence in intervening, and reduces negative attitudes toward people with mental illness (14, 15).

The relevance of MHFA, as a step toward long-term solutions for mental health care, is especially strong in Houston, one of the most diverse metropolitan areas in the United States. Nearly one-third of Houston’s residents are foreign-born, and the city is home to large Hispanic, Black, and Asian communities that often face persistent inequities in access to care (16). Structural barriers, such as language differences, stigma surrounding mental illness, limited insurance coverage, and a lack of culturally tailored services, make accessing professional mental health care especially challenging (17). Tailoring MHFA to these populations can help to overcome these barriers. For example, bilingual MHFA training and programs facilitated by trusted community leaders can increase accessibility, reduce stigma, and strengthen local support networks (18, 19). These adaptations not only strengthen community resources but also ensure that those most at risk are empowered to benefit from early intervention, positioning MHFA as a particularly appropriate strategy for Houston’s medically underserved and under-resourced communities.

Lying on the Gulf of Mexico, Houston has been subject to numerous disaster events, such as hurricanes and major flooding, over the past century, with several hurricanes causing billions of dollars in damages (20). The city’s geographic vulnerability to these types of extreme weather events makes it a particularly critical site for MHFA initiatives. Prior research has linked the impact of extreme weather events to worsened mental health outcomes, particularly perceived stress, general psychological distress, and posttraumatic stress (21). Raker and Smiley’s work supports these findings in the aftermath of Hurricane Harvey, a disaster that particularly impacted the Houston area in 2017, revealing that residents who experienced housing damage reported mental health distress at rates nearly three times higher than those whose homes remained intact (22). Bozick’s analysis of the physical and mental health of residents following Harvey reported a similar three-fold increase in poor mental health days among those with housing damage, with reports of diminished mental health more evident in parts of Houston where structural damage from Harvey was more severe (23). Fussell and Lowe adopted the conservation of resources (COR) theory (24) to explain the relationship between disaster exposure and mental health. According to this framework, disaster-induced losses often lead to chronic or secondary stressors, such as housing damage, substandard living conditions, and disruptions in employment, healthcare access, and social support systems, which have been strongly associated with increased levels of stress and depression (25). Experts emphasize the importance of integrating mental health support into the design of effective disaster response and recovery systems.

The implementation of community-based MHFA programs can help community members recognize the early signs of mental health crises, make timely referrals, and reduce potentially preventable emergencies (11). Beyond early detection, MHFA reduces stigma, improves mental health literacy, and strengthens supportive social networks, creating environments where individuals feel more comfortable seeking care (7, 8). Using a qualitative approach, this study assessed the feasibility and acceptability of MHFA training for improving community mental health among Houston residents who have experienced repeated extreme weather events.

## Methods

### Participants

Participants were recruited from MHFA training programs conducted between February and September 2025. Training attendees were invited to complete an optional post-training interview to assess their perceptions of and the utility of the MHFA training, perceptions of mental health, and experiences accessing mental health resources. Attendees were from underserved communities in Houston, Texas, including Fifth Ward, Kashmere Gardens, Trinity Gardens, and Galena Park, all of which have weathered repeated disaster events in the past decade. Eleven (of 42) MHFA training participants completed the optional post-training evaluation 6–8 weeks after completing the course.

### Procedure

Recruitment materials were distributed by community partners and posted at MHFA events. Participants who were interested in participating completed either an electronic or a paper consent form. Participants were contacted 6–8 weeks after course completion, either via email or phone, to participate in an individual interview to discuss their perceptions of the MHFA course. Interviews were completed via Zoom or Teams, and all interviews were audio-recorded and automatically transcribed using the Zoom transcription feature. Participants provided informed written and verbal consent to participate in the study, be audio-recorded, and for the use of de-identified verbatim quotes to be used in the dissemination of findings.

### Measures

#### Interview

A semi-structured interview guide was developed for individual interviews based on the literature. Specifically, completed research on mental health in medically underserved communities and the provision of mental health first-aid courses were reviewed, and all qualitative questions utilized in previous studies were considered in the development of the current interview guide (26). The questions targeted the participants’ perceptions of their mental health, community mental health, and feelings and attitudes towards the MHFA course. All interviews were conducted at least 6 weeks after the completion of the MHFA training.

#### Mental health support scale

Additionally, our evaluation assessed the behaviors taught in the MHFA course using the mental health support scale (MHSS) developed by Morgan et al. (27). The MHSS demonstrates good reliability and validity and includes 12 items with yes or no responses, in which a greater number of yes responses indicates a higher application of MHFA behaviors.

#### Qualitative data analysis

All the interviews were transcribed verbatim. Automated Zoom transcriptions were reviewed for accuracy, and phone interviews were manually transcribed and double-checked by trained research assistants. Following transcription, the data were analyzed according to Braun and Clarke’s process and reporting guidelines for reflexive thematic analysis (28). Specifically, this involved becoming familiar with the data, coding the transcribed interviews, and generating and reviewing themes that were subsequently organized and reported.

#### Trustworthiness

Rigor is recognized as equally important in qualitative research as in quantitative research (29, 30); therefore, multiple methods of trustworthiness were employed. All authors actively engaged individually and as a group in the reflexivity process (31). Specifically, the primary data analysts completed positionality statements individually and then discussed and reflected on them collectively. Collaborative and critical reflective discussions were held throughout the analytical process. Additionally, investigator triangulation was employed to ensure that diverse perspectives contributed to all components of the study, and negative case analysis was conducted to ensure that all perceptions and positions regarding a topic were considered (32). Finally, persistent observation of the data was conducted (33).

## Results

A breakdown of the demographic details of the participants who completed the interviews is presented in **Table 1**. Ten of 11 study participants were female, about 55% indicated English as the primary language spoken at home, and over 90% identified as non-Hispanic Black or Hispanic.

**Table 1:** Participant characteristics.

Demographic variable		N (%)
Sex		
	Female	10 (90.9%)
	Male	1 (9.1%)
Marital status		
	Single	4 (36.4%)
	Married	4 (36.4%)
	Other	3 (27.3%)
Primary language spoken in the home		
	English	6 (54.5%)
	Spanish	5 (45.5%)
Ethnicity		
	Non-Hispanic White	–
	Non-Hispanic Black	3 (27.3%)
	Hispanic	7 (63.6%)
	Other/Unknown	1 (9.1%)
Total household income		
	<$25,000	6 (54.5%)
	$25,000–$50,000	4 (36.4%)
	Prefer not to say	1 (9.1%)
Highest level of education		
	Some high school	3 (27.3%)
	High School	4 (36.4%)
	Other	4 (36.4%)

### Results of the MHSS supportive behaviors reported by participants

**Table 2** shows the responses to the MHSS supportive behaviors reported by participants. Seven of 11 (63.63%) participants indicated that someone they knew well had developed a mental health problem or experienced worsening mental health problems in the past 12 months; this individual was most commonly reported to be a family member (45.45%). Of those, more than 85% indicated that they asked about thoughts of self-harm (85.7%), discussed privacy and confidentiality (85.7%), demonstrated active listening by restating or summarizing (85.7%), communicated clearly (85.7%), conveyed hope (85.7%), offered resources (85.7%), and discussed professional help options (85.7%). Three items were reverse-coded. Few respondents endorsed trying to solve problems for the individual (14.3%), while 57% reported telling them to “get their act together” and 57.1% reported attempting to cheer them up by minimizing the situation. Other supportive behaviors, such as asking about other supportive people (71%) and discussing self-help strategies (71%), were frequently reported.

**Table 2:** Responses to the MHSS: supportive behaviors reported by participants, with reverse-coded items indicated (7 of 11 participants).

Based on your response that you know someone who developed a mental health problem or worsening mental health in the past 12 months, please indicate whether you:	Percentage of responses indicating respondent, provided support
1. Asked them whether they had thoughts of harming themselves or others.	6 of 7 (85.7%)
2. Discussed with them their wishes about privacy and confidentiality.	6 of 7 (85.7%)
3. Listened to their problems and tried to solve them.*	1 of 7 (14.3%)
4. Let them know you were listening to what they were saying by restating and summarizing what they have said.	6 of 7 (85.7%)
5. Communicated clearly and simply, and repeated things where necessary.	6 of 7 (85.7%)
6. Told them they had to get their act together.*	4 of 7 (57.1%)
7. Conveyed a message of hope by telling them help is available and things can get better.	6 of 7 (85.7%)
8. Tried to cheer them up by telling them that things do not seem that bad.*	4 of 7 (57.1%)
9. Offered them information and resources appropriate to their situation.	6 of 7 (85.7%)
10. Discussed their options for seeking professional help.	6 of 7 (85.7%)
11. Asked whether they had other supportive people they could rely on.	5 of 7 (71.4%)
12. Discussed with them whether they were interested in self-help strategies.	5 of 7 (71.4%)

* Indicates items that were reverse coded.

### Qualitative findings

Data analysis of the individual interviews generated three themes: (1) awareness and impact of mental health struggles and the role of the community, (2) understanding the realities of resource access and necessary steps to increase uptake, and (3) the utility and appropriateness of the mental health first aid program for underserved communities. The themes represent the collective stories of all the participants. Selected de-identified quotes that represent participant interviews are included; consent was obtained for the use of de-identified quotes. Interviews were conducted 6–8 weeks after completing the MHFA training.

### Theme 1: awareness and impact of mental health struggles and the role of the community

The first theme included discussions on individual and community mental health awareness. Responses included perceptions of how they perceive mental health and how it is perceived by their community. Furthermore, participant responses exploring the role of the community in mental health are included.

#### Subtheme 1: perceptions of individual mental health and the mental health of the community

Most participants noted that they did not previously have a thorough understanding of mental health; despite this, mental health was viewed in a positive light. Participants discussed how mental health was as important as physical health. Specifically, one participant voiced how “mental health, well, it’s like physical health right? It’s being able to manage your emotions in a way that doesn’t hurt you. Although sometimes it’s not possible because mental health is linked to physical health, a deficiency or something else. But when it’s a loss or something like that, then it can be managed by seeking help, and that’s how we move forward. If we have help in all possible ways, medical, spiritual, emotional, family, then we will achieve mental health” (MH017).

However, some participants did suggest that others may still view mental health through a lens of stigma.

Individual mental health was discussed differently by the participants. Challenges with mental health were described as transitional; that is, participants did not describe ongoing, debilitating mental health issues over long periods. Rather, participants described how they faced periods of difficulty with their mental health; “sometimes you isolate yourself from friends for the same reason, because you’re in a bad place mentally you know, you’re feeling down and you don’t know what to do” (MH009). Numerous factors were perceived to impact individual and community mental health. Specifically, personal factors such as physical challenges and family responsibilities were identified to impact one’s mental health. Some participants further described how they felt that the mental health of adults impacted the behavior and well-being of their children.

Specifically, one participant described how differences in perceptions of mental health and strategies to support mental health by different parents in different households impacted children’s mental health. This discrepancy in perceptions of mental health raised concerns among some participants that children’s mental health would be inappropriately handled. These concerns were detailed by one participant when discussing how parents of children with different opinions towards mental health were handling their child’s behaviors at a school: “so [the school] know that [the child] has a problem, and then [the father] fun up there and he tells [the school] his [child] don’t have a problem, all [the child] needs is a good whooping. [The child’s] having a problem and then you’re going to abuse him?” (MH008).

Societal factors were further highlighted as influencing mental health, and domestic and international news were highlighted as influential factors. Additionally, specifically within their community, participants described how successive disasters negatively impacted their community, and the fear associated with immigration changes contributed to poor mental health. When responding to societal impacts on their mental health, participants described how they would avoid daily exposure to protect their mental health:

“When you go through [ … ] things that happen in life, like storms, hurricanes, and all that, now that we’re dealing with immigration and everything, do you have mental health issues? Is your mind messed up? [ … ] Because you are [messed up], on no, I don’t watch TV anymore, because it’s all problems. I mean you focus on it, [and] your mind is messed up” (MH009).

#### Subtheme 2: importance of community connection: experiences with the mental health of others

The impact of poor mental health on all other aspects of one’s life was emphasized, and concern was expressed regarding how mental health challenges can lead to suicidal ideations or actions. Participants reflected on how their experiences with personal mental health challenges were perceived to negatively impact their relationships with others, both personally and professionally. The importance of establishing and maintaining community ties was perceived as critical for supporting one’s mental health. Many participants expressed how important their relationship with their community was, describing how they leaned on their neighbors for support and engaged with community organizations to stay informed. Leaning on members of their community was described by one participant who stated, “everybody talks to everybody and tells them, you need something, you can call me. It’s nice” (MH008). However, community connection was not experienced by all participants, as one stated, “here you kind of disconnect from the neighborhood, you know what I mean? [ … ] I live in a neighborhood that’s mostly Black [ … ] and you don’t have much connection with people, and here where I live, I don’t know them, I see them, but we haven’t had that great of a relationship” (MH017). While participants emphasized the support and information provided through social networks with strong community ties, it was not communally experienced.

### Theme 2: understanding the realities of resource access and necessary steps to increase uptake

The second theme centered around the resources perceived to be available for participants and their community. Specifically, discussions regarding the availability of resources and perceptions on the level of availability are explored. Further, the barriers to accessing services and utilized methods of coping are described.

#### Subtheme 1: mental health resource availability in the community

Participants expressed frustration that there were no known mental health resources available within their communities. This perception was articulated by one participant who said, “Well, there’s nothing around here, I mean there’s nothing around where I live. You have to go further away for medical resources” (MH017). Participants felt limited in their ability to support other community members experiencing mental health challenges and requiring professional support when no accessible resources were available. The perception that there were no easily accessible mental health resources was identified as an important factor contributing to negative overall mental health.

Furthermore, the lack of available trained mental health professionals was perceived to be harmful to the community and to lead to inappropriate reactions to those experiencing mental health crises. Specifically, one participant described how “someone happened to lose their life because they were in a crisis. The police answered the call, and it wasn’t dealt with the way it should have been dealt with” (MH012). Participants also felt that when resources were made available to the community, they were not always distributed or used appropriately.

#### Subtheme 2: understanding the barriers to accessing mental health support in underserved communities

Participants highlighted how unmet basic needs, such as food, water, and safe housing, constrain their ability to seek formal mental health services. Therefore, maintaining the fundamental needs for survival was prioritized over engaging with formal mental health services, as their financial and housing realities required them to focus first on immediate necessities, even though these basic needs were deeply intertwined with their overall mental well-being. This perspective was described by one participant who stated, “It’s not that they don’t care for mental health. They care for mental health, but it’s just one, we don’t have time, and two, it’s not affordable. We’re too busy trying to keep the lights on, the gas on, the water on, clothes on our backs, gas in the car, that type of deal” (MH002).

Financial constraints were further perceived as a barrier due to the location of mental health resources; the distance and time required additional funds that the participants did not have. Furthermore, participants described how fear surrounding their immigration status prevented individuals from seeking out and/or utilizing mental health resources. When discussing barriers to members of their community seeking out resources, one participant described how “the problem they have is that they also don’t have papers or anything like that, they’re afraid too; you understand me, they’re afraid” (MH002).

#### Subtheme 3: methods of coping with mental health challenges

Participants largely did not identify seeking professional mental health support due to barriers in accessing them, instead describing alternative methods of coping with mental health challenges and distress. Specifically, they described relying on spiritual beliefs, religion, family, and alternative self-care methods. The importance of religion was emphasized by the participants, who expressed that being actively involved in their religion positively impacted their mental health and helped them cope with mental health challenges. Participants described how, when they experienced mental health crises and did not feel as though there were any resources or support available, religion and the church were a safe place to turn. Religion was perceived to be a critical avenue of support because there was no location requirement; that is, participants could lean on their religion without leaving their homes through prayer. Participants also spoke about leaning on family and close friends to support their mental health. Specifically, participants described how they relied on their family and friends when they experienced mental distress; one participant voiced how “being united and being with them [ … ] that they come to the house, that they are here, that they stay here with you. That helps a lot. When my husband died, [family] supported me a lot and stayed here with me and [ … ] that helps you not to be depressed anymore” (MH007). Some participants described alternative coping mechanisms that they relied on to support their mental health. Alternative coping activities included spending time outside in green spaces, walking, and participating in physical activities such as gym classes (e.g., Zumba). Engaging in forms of self-care was perceived as helpful in supporting their mental health.

### Theme 3: the utility and appropriateness of the mental health first aid program for underserved communities

The final theme included discussions regarding the participants’ perceptions of the MHFA program. Furthermore, the perceived appropriateness and applicability of the program and the taught components were discussed. Finally, the long-term impact and utilization of the program were explored.

#### Subtheme 1: community perceptions of the mental health first aid program

MHFA was generally perceived positively by participants. Participants felt that it included relevant information and useful strategies for supporting those experiencing mental health crises and useful methods of self-care. Participants discussed their motivation to attend the MHFA program to learn more about mental health conditions and challenges. In fact, participants articulated that they felt that the program met or exceeded their expectations when they signed up. Furthermore, the positive perceptions of the program led participants to express their desire for an expansion of the program and additional events within their community. Positive feelings towards the MHFA program led to participants telling friends and other community members about the program and recommending attendance. Participants voiced that their biggest takeaway from the program was the increased knowledge surrounding mental health and the associated courage that knowledge gave them to support themselves and others. The perceived benefits of the program were further felt to benefit others; participants discussed how they had promoted the program and its benefits during their interviews.

#### Subtheme 2: understanding the appropriateness of MHFA for underserved communities in Houston, Texas

Participants detailed specific factors they liked in the MHFA program, factors that they disliked, as well as aspects that need to be integrated to improve the program for underserved communities such as theirs. There was a deep level of appreciation conveyed regarding the mental health education they received: “I loved it” (MH006). Some participants described how they obtained new knowledge on mental health, while others discussed how the program was a good refresher on mental health information that they had some pre-existing knowledge of from previous education or work. For example, one participant described how they had “been in the medical field for over 40-something years, and I have family members that suffer from mental health, so I think that it’s wonderful to continue to get updates on information that’s helpful to myself and my family” (MH012). A byproduct of the new and/or increased knowledge surrounding mental health was reported to be increased confidence and courage to be a vocal advocate and support for individuals experiencing mental health challenges. Furthermore, participants expressed feeling more comfortable acknowledging the challenges they were experiencing and recognizing when they should seek help. Specifically, one participant explained how the MHFA program “helped me a lot to feel a little more confident to say, *if I have this, I’ll go here*” (MH017). The active nature of the program, in which scenarios were discussed and participants were encouraged to ask questions, was further positively reviewed. Participants felt that this encouraged knowledge retention and a thorough understanding of the topics introduced throughout the course. Participants further appreciated the provision of materials on mental health first aid that they could take home, review, and use. They felt that the handbook provided with all of the discussed information was “very informative (MH005).

The information taught about mental health and the scenarios that were utilized to exemplify mental health challenges and strategies to support those in mental health crises were perceived to be appropriate for the community. Participants felt that “you could see yourself in [the] scenarios and then in the curriculum. It was able to draw things out that maybe sometimes you didn’t want to say” (MH011). Furthermore, participants described how their interactions throughout the class directly combatted stigma. Specifically, one participant said, “I think the stigma is off for them, and they were just empowered to do something for themselves because everyone needs a place where they can go to feel heard, and they felt heard [ … ] that took the stigma off of it for them” (MH011).

Notably, while some participants felt that the language used during the program was inclusive and understandable, others felt that there was unnecessary jargon that some participants may not comprehend. Participants made suggestions to improve the program and ensure its inclusivity for underserved communities, such as their own. A salient aspect of the program that could be improved is the language in which it is provided. Specifically, some participants advocated for the program to be provided in Spanish to be inclusive of participants who felt more comfortable learning in Spanish as opposed to English. Furthermore, in addition to discussing different resources and advocating for their utilization, some participants felt that specific contact information and guidelines regarding the path to access them would be helpful.

Furthermore, although the scenarios discussed were perceived as appropriate and conducive to participant comprehension, some participants suggested including active engagement in the form of acting out scenarios to further improve understanding, comfort, and confidence in real-life situations. One participant described how an included portion of the program could “have [scenarios] acted out so people would [ … ] know the situations that they encounter with people, and that way they will know how to react or know that person has mental problems” (MH005). Scenarios that were perceived to be missing included interacting with a family member during a mental health crisis. Participants articulated a desire to understand how to navigate those scenarios: “more on different type[s] of crises that family members may face with having family members that suffer from mental health, the way to deal with it before they call the police” (MH012).

Moreover, discussion and topics surrounding LGBTQ issues were perceived to be missing from the training. That is, some participants felt that “they didn’t talk about [ … ] LGBT [ … ] they could include that so people can feel more confident because a lot of people think that they shouldn’t say they’re gay and they don’t come out [of] the close because they think that it’s bad and I don’t think it’s bad” (MH008). Notably, some concerns regarding approaching situations in which mental health crises in minors were discussed among adults were identified. Participants were unsure how to best approach these scenarios to support the individual while managing concerns over government involvement, such as child protective services.

While participants largely approved of and enjoyed the content that was presented, some participants desired an extension of the program. Specifically, the participants felt that the opportunity for additional individual reflection and discussion of the program was extremely beneficial. While participants recognized that the individual interviews were a separate research project on the MHFA program, some felt that individual post-program discussions should be incorporated into the program. The value of having the opportunity to discuss the information taught in the MHFA program and their perceptions regarding its importance was described by one participant, who stated, “with the research thing, the one-on-one, the one-on-one is life-changing. It gives you a change to really be present. It gives you a change to just get it off your chest” (MH011). Another participant described how “it’s been a source of relief to be able to talk to you this morning, especially about a lot of the things that I’ve said” (MH013). Furthermore, some participants felt that it was a lot of information at once, and at times, they felt it was difficult to keep up with the topics being covered. That is, the topics covered were perceived to occasionally jump around, in which one topic was reviewed and, then the discussion jumped to another topic, which some participants felt made it difficult to follow and keep up with the information presented. Some participants faced challenges in following the presentation; they felt that “there were parts that we did understand and parts that left us in limbo” (MH009). Relatedly, some participants stated that their preferred language was Spanish and desired that the course be available in different languages. Furthermore, while the content was perceived as helpful, the length of a single session was not perceived to be optimal. That is, participants suggested that a full-day session was a long period of time to remain focused and questioned whether the session could be organized differently to decrease the time of the program while maintaining the content. One participant suggested that the program be “split up over different days (34) more time, and some people like a little homework” (MH011). Participants felt that adjusting the presentation organization of the program may encourage their peers to engage more in the program, as they didn’t like that “a lot of [others in the class] were standing up and not paying attention anymore” (MH006).

#### Subtheme 3: long-term impacts of the MHFA strategies

The participants reported the continued use of the strategies and concepts taught in the MHFA program. The information provided and strategies taught were identified by participants as being useful and valuable for future interactions related to mental health. While not all participants described scenarios in which they interacted with others experiencing mental health crises, some participants described the utility of the taught strategies and the confidence instilled by knowing them. Specifically, participants felt capable and confident in helping others after completing the course.

While participants described how they had some existing knowledge and/or preconceptions regarding mental health, the course provided them with knowledge and strategies that they found to be applicable and useful. A key strategy identified by participants as important and remaining salient in their memory was communication. Participants described how they consciously integrated the communication strategies taught through the ALGEE framework to organize mental health discussions with others. The course was perceived to prepare participants for real-life situations in which they or others may experience a mental health crisis: “I do feel prepared to be honest [ … ] much more prepared, much more because now I have the resources to offer” (MH017). Participants further described how they felt they were giving back to their community by completing this course and obtaining the knowledge and strategies. Specifically, the participants felt confident in their ability to help individuals experiencing mental health crises within their community. Participants articulated how “if I would see somebody that they were trying to kill themselves or something, I would now know what to tell them” (MH008). They were confident in their ability to engage with individuals in crises and introduce available resources (e.g., mental health practitioners/resources) to them and/or contact emergency services. The confidence provided to participants through the knowledge and strategies taught was positively perceived across participants: “I always felt that I could and I would [help], and [the course] just reassured me that I could help someone and direct them to get help and to listen” (MH012). Participants further reflected on the course’s perceived long-term impact on their community. Specifically, participants felt that the benefits of the course would positively impact their community through the spread of knowledge and support for mental health. When discussing this impact, one participant explained how they “think [the course] was very helpful for the community. Everyone that came, they still talk about it, they want more” (MH011).

Finally, participants referenced the importance of self-care throughout the MHFA program. That is, they described their continued attention to including self-care in their routines and schedules. Furthermore, more importance was placed on prioritizing self-care to maintain their mental health than prior to attending the MHFA training. Different methods of self-care were described across participants, one of which included “going to a community garden and there I feel like I relax [ … ] touching the earth, moving plants, watering plants” (MH017). Participants reported that they consciously ensured that they integrated self-care into their schedules and prioritized their own mental health. Additionally, participants described how they discussed the importance of self-care in community organization meetings. This has reportedly resulted in the integration of self-care within community organizations for community members.

## Discussion

This study examined the acceptability and utility of MHFA training for disaster preparedness in underserved disaster-prone communities. Overall, we found the training to be useful and well received, with participants demonstrating largely correct application of key skills following the training. Past research on the effectiveness and applicability of the training has been conducted in study populations that are largely homogenous, with high education levels, or are in a workplace cohort (34–36). However, this training was geared towards lay communities and may be especially beneficial to underserved populations; therefore, this study was critical to better understand the perceived appropriateness and utility of this program by members of these communities. Participants’ perceptions of mental health varied in importance, with some viewing it as equal to physical health. This variation among the study participants could indicate the decreasing stigma surrounding mental health over the past 20 years (37). Poor mental health was also viewed by participants as a continually fluctuating state, wherein an individual can experience different degrees of mental wellness over time, which may be influenced by external stressors, including governmental policy changes and natural disasters. This view corresponds with research showing that increased media exposure to the reporting of world events increases stress levels and poorer mental health (38, 39).

Additionally, the interviewees highlighted the perceived lack of accessible mental health resources in their communities. This finding supports previous research that noted accessibility and cost as barriers to initiating treatment for mental health issues (10). Participants also noted the lack of monetary resources available for mental health treatment, noting that other needs were prioritized. Examples included providing for the basic physiological needs of the participant and their family, including paying for electricity, water, gas services, clothing, and food.

Overall, participants had a positive response to the MHFA training and felt that they were more equipped to help individuals in their community, which echoes the findings of both Kitchener and Jorm and Bahn et al., who performed program evaluation and found that individuals reported significant increases in their knowledge, helping behavior, and confidence in helping others (35, 40). The findings also highlighted participants’ perception of the program’s appropriateness for their community, as many found that the current language and delivery of the program were appropriate for their community. This sentiment does not entirely support previous research, which found that tailoring the program to be culturally appropriate was beneficial to the delivery and acceptance of the program (41). Specifically, the findings suggest that the existing MHFA program was considered appropriate and acceptable to participants from different communities and cultures.

### Limitations and future directions

This study has some limitations. Due to the small sample size, the findings may not be generalizable to other disaster-prone communities. Student participants self-selected to attend the MHFA training, which may indicate a pre-existing interest in mental health issues. This could introduce selection bias, as participants may be more motivated or engaged than the broader student population, potentially influencing the generalizability of our findings. Furthermore, participants were interviewed 6–8 weeks after completing the MHFA program, and it is plausible that the long-term impact and utility of the information and strategies taught in the MHFA had not yet been used by participants. Therefore, future longitudinal research is necessary to assess the possible long-term implications of individual mental health perceptions and responses within the community following the completion of the MHFA program. Finally, the interviews were conducted entirely virtually; there may be different perspectives between individuals who are comfortable using telecommunication and those who would prefer in-person discussions.

## Conclusion

The MHFA program was perceived as valuable, empowering, and applicable, enhancing participants’ knowledge, confidence, and ability to support others in mental health crises. These findings underscore the potential of community-based mental health training to strengthen individual and collective resilience in underserved and disaster-prone communities.

## Data Availability

The datasets presented in this article are not readily available because they include confidential information that identifies the participants. Requests to access the datasets should be directed to Dr. Adepoju, oadepoju@uh.edu.
